# Draft Genome Sequence of *Streptomyces mutabilis* TRM45540, Isolated from a Hypersaline Soil Sample

**DOI:** 10.1128/genomeA.01465-15

**Published:** 2015-12-17

**Authors:** Xiaoxia Luo, Chuanxing Wan, Lili Zhang

**Affiliations:** College of Life Science, Key Laboratory of Protection and Utilization of Biological Resources in Tarim Basin of Xinjiang Production & Construction Corps, Tarim University, Alar, China

## Abstract

We report here the draft genome sequence of *Streptomyces mutabilis* TRM45540, a strain isolated from a soil sample from Xinjiang, China. Analysis of the genome using the bioinformatics tool antiSMASH showed the presence of many unique natural-product biosynthetic pathways.

## GENOME ANNOUNCEMENT

*Streptomyces mutabilis* TRM45540 was originally isolated from a soil sample from Loulan in Xinjiang, China (89°22′22″E 40°29′55″N). It produces the antibiotic actinomycin D, which is active against a broad range of pathogens, such as the pathogens of cotton, *Fusarium oxysporum* f. sp. *vasinfectum* and *Verticillium dahlia* Kleb, for which the diameter of the inhibition zone was 83 mm.

The genome of *S. mutabilis* TRM45540 was sequenced with a mixed strategy using an Illumina HiSeq 2000 platform. Two different sequencing libraries, a 500-bp paired-end and 6,000-bp mate-pair library, were used. Sequencing yielded 230-fold genome coverage. Assembly of all sequence reads (1,002-Mbp paired-end reads and 808-Mbp mate-pair reads) applying the SOAP*denovo* 1.05 software (1) resulted in a draft genome comprising 39 scaffolds, with 41 contigs with an *N*_50_ size of 2,626,853 bp and a longest contig of 3,410,897 bp. The total scaffold size was determined to be 7,828,944 bp, with a G+C content of 71.6%. Genome annotation was accomplished using the Rapid Annotations using Subsystems Technology (RAST) server (2). Briefly, protein-coding genes were identified using Glimmer version 3.0 (3). Ribosomal RNAs were predicted by a sequence similarity search using BLAST against an RNA sequence database and/or using the Infernal and Rfam models (4, 5). Transfer RNAs were predicted using tRNAscan-SE (6). The predicted coding sequences (CDSs) were translated and used to search the National Center for Biotechnology Information (NCBI) nonredundant (NR), UniProt, TIGR-Fam, Pfam, PRIAM, KEGG, COG, Swiss-Prot, TrEMBL, GO, and InterPro databases. Other noncoding genes were predicted using Infernal 1.0 (7). Additional gene prediction analysis and functional annotation were performed within the Integrated Microbial Genomes-Expert Review (IMG-ER) platform (8). Gene prediction revealed 7,526 protein-coding genes, 6 rRNA operons, and 43 tRNA genes. Among these protein-coding sequences, 5,408 (71.86%) have been assigned to a COG functional category (9).

The complete genome sequence includes a number of gene regions devoted to the biosynthesis of secondary metabolites. Scanned by antiSMASH (10), we found 6 type I, 2 type II, and 1 type III polyketide biosynthetic gene clusters and 2 nonribosomal peptide synthetase biosynthetic gene clusters. Also, we found 5 gene clusters for a terpenoid-like bioactive substance, which possess significant antibiotic properties and cancer cell cytotoxicities (11).

The *S. mutabilis* TRM45540 genome sequence provides us the opportunity to further understand the evolutionary relationship between *Streptomyces* strains, to explain the physiological mechanism for its growth in hypersaline soil, and to screen more active metabolites applied in agriculture and pharmacy.

### Nucleotide sequence accession number.

The *S. mutabilis* TRM45540 draft genome sequence was deposited in the NCBI database under the accession no. JNFQ00000000. The version described in this paper is the first version.

## References

[1] Li R, Zhu H, Ruan J, Qian W, Fang X, Shi Z, Li Y, Li S, Shan G, Kristiansen K, Yang H, Li S, Wang J, Wang J 2010 *De novo* assembly of human genomes with massively parallel short read sequencing. Genome Res 20:265–272. doi:10.1101/gr.097261.109. 20019144 PMC2813482

[2] Aziz RK, Bartels D, Best AA, DeJongh M, Disz T, Edwards RA, Formsma K, Gerdes S, Glass EM, Kubal M, Meyer F, Olsen GJ, Olson R, Osterman AL, Overbeek RA, McNeil LK, Paarmann D, Paczian T, Parrello B, Pusch GD, Reich C, Stevens R, Vassieva O, Vonstein V, Wilke A, Zagnitko O 2008 The RAST server: Rapid Annotations using Subsystems Technology. BMC Genomics 9:75. doi:10.1186/1471-2164-9-75. 18261238 PMC2265698

[3] Delcher A, Harmon D, Kasif S, White O, Salzberg SL 1999 Improved microbial gene identification with Glimmer. Nucleic Acids Res 27:4636–4641. doi:10.1093/nar/27.23.4636. 10556321 PMC148753

[4] Griffiths-Jones S, Bateman A, Marshall M, Khanna A, Eddy SR 2003 Rfam: an RNA family database. Nucleic Acids Res 31:439–441. doi:10.1093/nar/gkg006. 12520045 PMC165453

[5] Eddy SR 2002 A memory-efficient dynamic programming algorithm for optimal alignment of a sequence to an RNA secondary structure. BMC Bioinformatics 3:18. doi:10.1186/1471-2105-3-18. 12095421 PMC119854

[6] Lowe TM, Eddy SR 1997 tRNAscan-SE: a program for improved detection of transfer RNA genes in genomic sequence. Nucleic Acids Res 25:955–964. doi:10.1093/nar/25.5.0955. 9023104 PMC146525

[7] Nawrocki EP, Kolbe DL, Eddy SR 2009 Infernal 1.0: inference of RNA alignments. Bioinformatics 25:1335–1337. doi:10.1093/bioinformatics/btp157. 19307242 PMC2732312

[8] Markowitz VM, Mavromatis K, Ivanova NN, Chen IA, Chu K, Kyrpides NC 2009 IMG ER: a system for microbial genome annotation expert review and curation. Bioinformatics 25:2271–2278. doi:10.1093/bioinformatics/btp393. 19561336

[9] Kristensen DM, Kannan L, Coleman MK, Wolf YI, Sorokin A, Koonin EV, Mushegian A 2010 A low-polynomial algorithm for assembling clusters of orthologous groups from intergenomic symmetric best matches. Bioinformatics 26:1481–1487. doi:10.1093/bioinformatics/btq229. 20439257 PMC2881409

[10] Blin K, Medema MH, Kazempour D, Fischbach MA, Breitling R, Takano E, Weber T 2013 antiSMASH 2.0—a versatile platform for genome mining of secondary metabolite producers. Nucleic Acids Res 41:W204–W212. doi:10.1093/nar/gkt449. 23737449 PMC3692088

[11] Soria-Mercado IE, Prieto-Davo A, Jensen PR, Fenical W 2005 Antibiotic terpenoid chloro-dihydroquinones from a new marine actinomycete. J Nat Prod 68:904–910. doi:10.1021/np058011z. 15974616

